# Supplementary material for: SimpleMind: An open-source software environment that adds thinking to deep neural networks
Source: PLoS One. 2023 Apr 13;18(4):e0283587. doi: 10.1371/journal.pone.0283587 (PMC10101376; doi:10.1371/journal.pone.0283587)
Supplement: Attachment — Submitted filename: comments_01012023.pdf [file pone.0283587.s001.pdf]

The authors developed a software framework called “SimpleMind” for reliable medical image analysis. One extremely interesting aspect of the SimpleMind framework lies in its ability to incorporate and utilize domain knowledge, which is important for analyzing different types of medical images [1]. This paper is very well written, the experiments are comprehensive, and its technical contributions are solid. Thank the authors for this amazing work!  I look forward to seeing further extensions and applications of this framework on various medical imaging tasks, like classification and detection in the future.

Just one minor comment: Figure 2, 4, 7, 11, 13 are blurry, making texts the image captions unreadable. I guess this is because the journal’s submission system failed to preserve the original resolutions. Please make sure higher-resolution versions are used for all the figures when it comes to publishing.

[1] "Recent advances and clinical applications of deep learning in medical image analysis." Medical Image Analysis (2022).
