# Supplementary material for: SimpleMind: An open-source software environment that adds thinking to deep neural networks
Source: PLoS One. 2023 Apr 13;18(4):e0283587. doi: 10.1371/journal.pone.0283587 (PMC10101376; doi:10.1371/journal.pone.0283587)
Supplement: Attachment — Submitted filename: Response to Reviewers.pdf [file pone.0283587.s002.pdf]

Response to Reviewers

Thank you for providing us with careful and constructive reviews and for your helpful comments. We appreciate the possibility to revise our initial submission.
We revised the manuscript to address all the points raised. All edits in the manuscript have been highlighted, and the numbered responses are provided below. The review comments are shown in italics and the corresponding answer to each comment are followed.

Response to the Journal Requirements

1. Please ensure that your manuscript meets PLOS ONE's style requirements, including those for file naming. The PLOS ONE style templates can be found at https://journals.plos.org/plosone/s/file?id=wjVg/PLOSOne_formatting_sample_main_body.pdf and https://journals.plos.org/plosone/s/file?id=ba62/PLOSOne_formatting_sample_title_authors_affiliations.pdf

For the journal requirements, we edited the manuscript to meet the style requirements including the file naming.

2. *Please note that PLOS ONE has specific guidelines on code sharing for submissions in which author-generated code underpins the findings in the manuscript. In these cases, all author-generated code must be made available without restrictions upon publication of the work. Please review our guidelines at https://journals.plos.org/plosone/s/materials-and-software-sharing#loc-sharing-code and ensure that your code is shared in a way that follows best practice and facilitates reproducibility and reuse.*

We checked our public code repository with the guidelines.

3. We noted in your submission details that a portion of your manuscript may have been presented or published elsewhere. [A preprint of this article was uploaded to the arXiv archive: https://arxiv.org/abs/2212.00951] Please clarify whether this publication was peer-reviewed and formally published. If this work was previously peer-reviewed and

published, in the cover letter please provide the reason that this work does not constitute dual publication and should be included in the current manuscript.

This manuscript is a first submission of the preprinted version uploaded in the arXiv archive. The arXiv version of the manuscript has not been peer-reviewed nor formally published before this submission. The format of the preprint changed for this submission, but the context remains the same. We believe that the PLOS One journal allows authors to post a manuscript on a preprint server. (https://journals.plos.org/plosone/s/submission-guidelines#loc-related-manuscripts)

4. In your Data Availability statement, you have not specified where the minimal data set underlying the results described in your manuscript can be found. PLOS defines a study's minimal data set as the underlying data used to reach the conclusions drawn in the manuscript and any additional data required to replicate the reported study findings in their entirety. All PLOS journals require that the minimal data set be made fully available. For more information about our data policy, please see http://journals.plos.org/plosone/s/data-availability. Upon re-submitting your revised manuscript, please upload your study's minimal underlying data set as either Supporting Information files or to a stable, public repository and include the relevant URLs, DOIs, or accession numbers within your revised cover letter. For a list of acceptable repositories, please see http://journals.plos.org/plosone/s/data-availability#loc-recommended-repositories. Any potentially identifying patient information must be fully anonymized.

Important: If there are ethical or legal restrictions to sharing your data publicly, please explain these restrictions in detail. Please see our guidelines for more information on what we consider unacceptable restrictions to publicly sharing data:http://journals.plos.org/plosone/s/data-availability#loc-unacceptable-data-access-restrictions. Note that it is not acceptable for the authors to be the sole named individuals responsible for ensuring data access. We will update your Data Availability statement to reflect the information you provide in your cover letter.

The public prostate segmentation dataset PROMISE12 (https://promise12.grand-challenge.org) is a minimal data set underlying the prostate result demonstrating the effectiveness of the SimpleMind environment. The result is fully reproducible with the public SimpleMind prostate application (https://gitlab.com/sm-ai-team/simplemind-applications/-/tree/develop/mri_promise12_quick_start) using PROMISE12 dataset only. We included the information in the revised cover letter as well.

Response to Reviewer 1

This paper proposes an open-source software framework, i.e., SimpleMind, for explainable AI in medical image analysis. Since the “black-box“ property of deep neural networks greatly hinders its application in medical image analysis, how to enhance the interpretability and build trustworthy AI system is an important topic. Thus the idea and motivation of this work are good. The authors use three experiments with different modalities (i.e., CT, MRI, X-ray) to illustrate the usage of the software, and verify its effectiveness in medical image analysis.There are several comments, concerns, and suggestions for this work.

Thank you for your thoughtful comments. We appreciate your positive feedback about our idea and motivations. We edited the manuscript to address your comments and improve the paper. All edits in the manuscript have been highlighted, and the numbered responses are provided below.

[1] Since this is a software paper rather a technical/theoretical paper, it is important to clearly illustrate the overall structure design of this software. The current version is not good enough on this point. What’s the input/output of the software? How many modules and what are their roles?

Thank you for this valuable comment, it has helped us strengthen the submission as a software paper. We have made substantial edits to the “Open Source Implementation” to describe the software environment, its modules and their roles from a developers perspective.

We also made edits throughout the manuscript for clarity and consistency of terminology. We have revised “framework” to “environment” based on the functional scope of the SimpleMind software. Within the SimpleMind environment, users can apply (run) an existing knowledge base (application) to process an input image, they can create a new knowledge base, they can initiate learning to update a knowledge base, or extend its modules by programming the open source. Edits have been made throughout the paper to reflect these capabilities. In particular, we have restructured the “Open Source Implementation” section to become a subsection of a new "SimpleMind Software Environment" section that describes the Think and Learn modules. It now clearly describes the roles and inputs/outputs of these modules.

[2] As a software paper, a detailed description of the workflow of the software is essential. For a reader who is interested in this work, how to use the software step-by-step? Does this software have a user-interface? Adding a section about this content is needed.

As described above, a new "SimpleMind Software Environment" section describes the Think and Learn modules as well as creation of a knowledge base. It explains how a user interacts with each model to create and then optimize a knowledge base in SimpleMind. We also expanded the 2nd last paragraph of the Introduction to give an overview of two types of user interaction with the environment. We again thank the reviewer for their comments which have led to substantial revision and improvement of the paper.

[3] Currently, interpretable AI has become a very hot topic. As for DNN, many works such as attention mechanism have greatly enhanced the interpretability of DNNs. What's the advantage (or differences) of this work over these methods (e.g. attention techniques in DNNs)?

SimpleMind supports DNNs by embedding DNN nodes within a knowledge base, so it is complementary rather than competing with existing methods for DNN attention and interpretability. When the Think module is run, it writes out the content of the Blackboard which contains all of the byproducts from pre-processing the inputs for DNN nodes, training the DNN weights, post-processing the outputs of DNN nodes and selecting the final results with consistency checks (whether the predictions are matched with the expected ranges for characteristics and relationships between other anatomical objects), in a human-readable way. This includes the image search area for a DNN node that is derived from spatial relationships with other anatomical objects and used to crop the image prior to input to the DNN. Thus, SimpleMind encompasses and exposes many elements of its decision making and may have more comprehensive interpretability than other systems. Its Blackboard is also human readable by a domain expert rather than only by a data scientist. Current techniques using attention maps alone are insufficient to explain how those maps arise. In contrast, the Blackboard allows for examination of related objects to see how the search area was computed, providing us with explanations beyond the DNN node itself. We address interpretability in Item #2 of the section on "Supporting and Improving Deep Neural Networks with

SimpleMind Reasoning” and have added a sub-bullet that uses attention as an example of interpretability via the Blackboard.

[4] The experiments lack comparisons with some baselines. For example, set a classic DNN (ResNet, UNet, …) as the baseline, then compare the proposed software with it. Showing some failure cases of baseline is helpful to illustrate the effectiveness of the proposed method.

We have added baseline comparisons with a single DNN using the same three-dimensional U-Net architecture, input pre-preprocessing and the same training procedures that the kidney and prostate applications used in their knowledge bases. We revised the application examples to illustrate how the proposed method improves the results from the baseline using failure case examples. We did not implement the baseline single DNN model for the chest X-ray application during its evaluation.

[5] The genetic algorithm (GA) has some randomness. Will this influence the result (output) of this software?

The trajectory of the GA optimization can be influenced by the starting seed points, which may lead to different optimized tunable parameter sets. In SimpleMind, the json configuration file for the GA training contains the seed number for controlling the randomness. With the same seed number, users will get the same GA chromosomes for the next generations. Users also specify the range of the possible values for the tunable knowledge base parameters (e.g. max thresholding, to be set between X and Y image intensity values), so the range of variation already constrains the optimization (and indirectly, the effect of randomness) within user-defined ranges. While the genetic algorithm is the initial method implemented as the optimizer employed in KNoLO, the KNoLO optimizer is modular and can be swapped with other optimization methods that may better fit particular user needs.

Reviewer’s Responses to Questions 3. Have the authors made all data underlying the findings in their manuscript fully available?The PLOS Data policy requires authors to make all data underlying the findings described in their manuscript fully available without restriction, with rare exception (please refer to the Data Availability Statement in the manuscript PDF file). The data should be provided as part of the manuscript or its

supporting information, or deposited to a public repository. For example, in addition to summary statistics, the data points behind means, medians and variance measures should be available. If there are restrictions on publicly sharing data—e.g. participant privacy or use of data from a third party—those must be specified. "No"

We have added information about the training dataset and process for the three applications. We also defined our minimal data set as a public dataset PROMISE12 for prostate segmentation, which shows the effectiveness of the SimpleMind environment. The findings in the prostate applications are fully available without restriction. Portions of the data used for kidney and chest x-ray applications are collected from UCLA and restricted for sharing. We specified the restrictions on UCLA data in the manuscript.

Response to Reviewer 2

The authors developed a software framework called "SimpleMind" for reliable medical image analysis. One extremely interesting aspect of the SimpleMind framework lies in its ability to incorporate and utilize domain knowledge, which is important for analyzing different types of medical images. This paper is very well written, the experiments are comprehensive, and its technical contributions are solid. Thank the authors for this amazing work! I look forward to seeing further extensions and applications of this framework on various medical imaging tasks, like classification and detection in the future.

We appreciate the reviewer's encouraging feedback, to provides motivation for future tasks and improvements. Your thoughtful comments remind us of the importance of domain knowledge. Our primary goal was to help end-users develop their application with domain knowledge without worrying about the processing code. We have revised the term software "framework" to "environment" to clarify the functional scope of the SimpleMind software. Users can build an application directly and completely from domain knowledge by just creating a knowledge base, and the SimpleMind environment provides a runtime Think module to apply it to understand an image.

Just one minor comment: Figure 2, 4, 7, 11, 13 are blurry, making texts the image captions unreadable. I guess this is because the journal's submission system failed to

preserve the original resolutions. Please make sure higher-resolution versions are used for all the figures when it comes to publishing.

Thank you for these observations. We corrected the Figure resolution issues and uploaded new versions.
